# Construction and Analysis of a Joint Diagnosis Model of Random Forest and Artificial Neural Network for Obesity

**DOI:** 10.3389/fmed.2022.906001

**Published:** 2022-05-23

**Authors:** Jian Yu, Xiaoyan Xie, Yun Zhang, Feng Jiang, Chuyan Wu

**Affiliations:** ^1^Department of Rehabilitation Medicine, The First Affiliated Hospital of Nanjing Medical University, Nanjing, China; ^2^Department of Neonatology, Obstetrics and Gynecology Hospital of Fudan University, Shanghai, China

**Keywords:** obesity, gene sequencing technology, random forest classifier, artificial neural network, diagnosis model

## Abstract

Obesity is a significant global health concern since it is connected to a higher risk of several chronic diseases. As a consequence, obesity may be described as a condition that reduces human life expectancy and significantly impacts life quality. Because traditional obesity diagnosis procedures have several flaws, it is vital to design new diagnostic models to enhance current methods. More obesity-related markers have been discovered in recent years as a result of improvements and enhancements in gene sequencing technology. Using current gene expression profiles from the Gene Expression Omnibus (GEO) collection, we identified differentially expressed genes (DEGs) associated with obesity and found 12 important genes (CRLS1, ANG, ALPK3, ADSSL1, ABCC1, HLF, AZGP1, TSC22D3, F2R, FXN, PEMT, and SPTAN1) using a random forest classifier. ALPK3, HLF, FXN, and SPTAN1 are the only genes that have never been linked to obesity. We also used an artificial neural network to build a novel obesity diagnosis model and tested its diagnostic effectiveness using public datasets.

## Introduction

Obesity, defined by the European Association for the Study of Obesity (EASO) (1), as an adiposity-related chronic illness, is a continuing global health concern because it is frequently linked to increased risks for a variety of chronic illnesses, including hypertension, type 2 diabetes (T2D), and cardiovascular disease (CVD). As a consequence, obesity may be described as a condition that reduces human life expectancy and significantly impacts life quality. Obesity has a complicated etiology, with environmental, social, physiological, medicinal, behavioral, genetic, epigenetic, and other variables all contributing to cause and development (2). Obesity has surged globally in the previous two decades, according to a study, and is spreading like an epidemic illness.

Obesity is categorized into two types: physical obesity and feeding obesity. Simple obesity seems to be the most prevalent kind. Secondary obesity is defined by excessive fat stores in the body, but it also has the clinical signs of primary illness. It is induced by hormonal or metabolic abnormalities. Drugs that cause gaining weight as a side effect are becoming more widely used, which contributes to drug-induced obesity. As a result, the therapies for these three forms of obesity are distinct. Obesity is traditionally treated with behavioral modification, medication therapy, and weight reduction surgery. Weight reduction surgery, which would be a risky invasive operation, is the only long-term therapy. Obesity and diabetes are now being treated using neuromodulation techniques which include vagal nerve stimulation as well as intestinal electrical stimulation.

Obesity diagnostic procedures that are routinely utilized have certain drawbacks. Currently, BMI (body mass index) is by far the most widely used metric for determining obesity. However, a BMI diagnosis alone will not be able to determine the site of fat distribution (3). The WHO included waist circumference as just a criterion of abdominal adiposity in its obesity categorization paradigm because it offered extra information about the risk of CVD as a consequence of the BMI category (4). It's worth noting that BMI, as well as waist circumference cut-offs, change by ethnicity since these measurements are associated with a higher risk of heart illness and diabetes in distinct ways (5–9). As a result, new diagnostic models must be developed to enhance current procedures.

**Figure 1:**
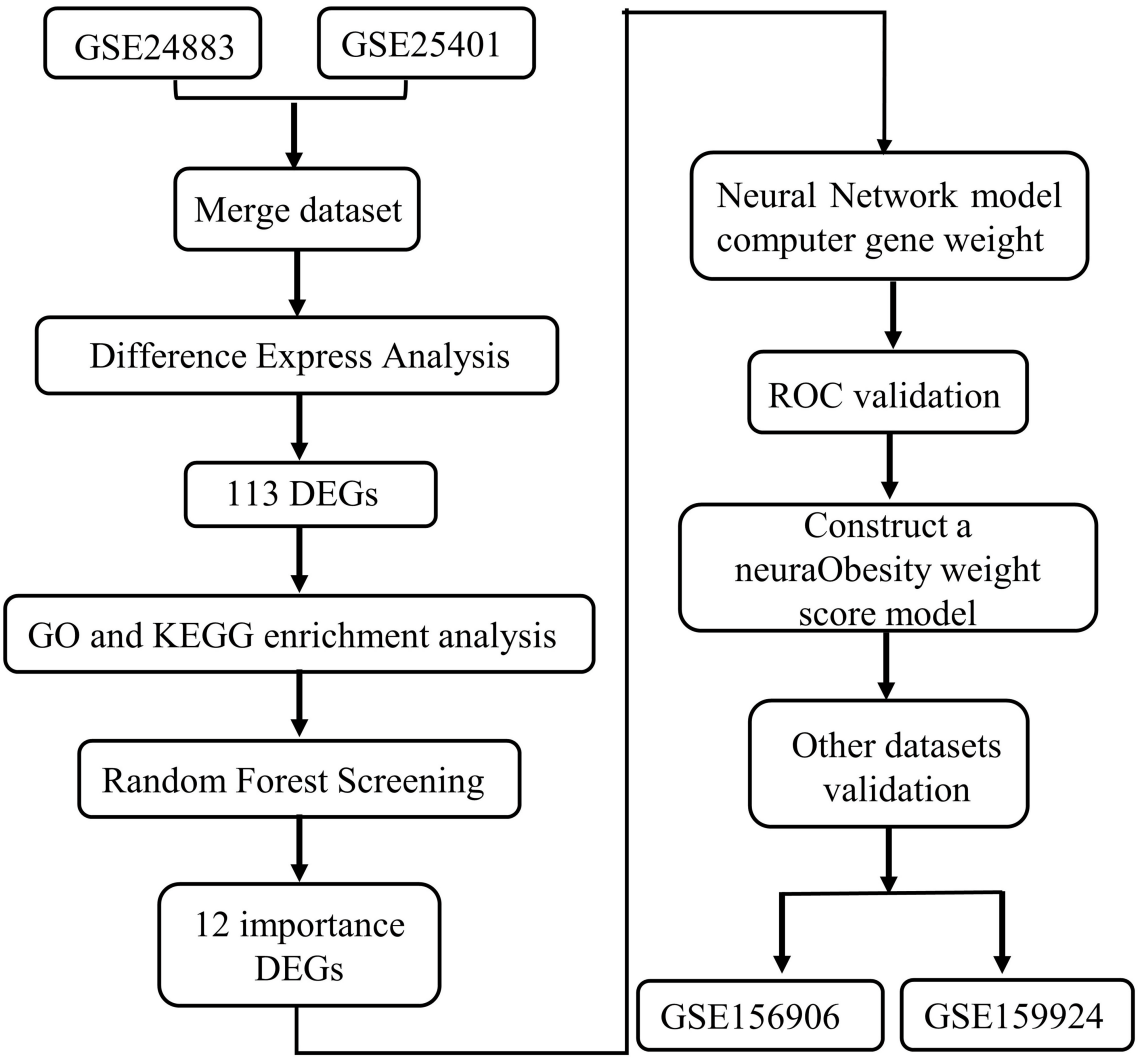
Flowchart.

The fast advancement of 2nd sequencing technology has aided in the discovery of marker genes linked to a wide range of disorders in recent years, laying a strong basis for the creation of a novel gene-related diagnostic approach for obesity. In this work, we searched the gene expression comprehensive database (GEO) for differentially expressed genes (DEGs) between obese patients' fat samples and normal fat samples. We apply the random forest approach to determine the important genes activated in obesity based on this DEGs data. Then, using an artificial neural network, we built a genetic diagnostic model of obesity based on these critical genes (see the analysis process in Figure 1).

## Materials and Methods

### Downloading and Analyzing Data

Gene Expression Omnibus (GEO, http://www.ncbi.nlm.nih.gov/geo) was used to find DEGs. The following were the selection criteria: Table 1 shows the expression pattern and clinical phenotypic data from chip datasets GSE24883, GSE25401, as well as RNA-seq datasets GSE156909 and GSE159924, that were downloaded using the query tool. The GEO database was used to collect the annotation data for the chip probes of the appropriate platforms. Multiple probes were identified to match one gene symbol during the translation of chip probe ID as well as the gene symbol. The median probe expression was taken as the level of gene expression in this situation.

**Table 1:** Data download.

**ID**	**GSE number**	**Data type**	**Samples**	**Source type**	**Group**
1	GSE24883	Microarray	8Lean8Obesity	subcutaneous adipose tissue	Discovery cohort
2	GSE25401	Microarray	26Lean30Obesity	subcutaneous adipose tissue	Discovery cohort
3	GSE156906	RNA-Seq	14Lean28Obesity	subcutaneous adipose tissue	Validation cohort
4	GSE159924	RNA-Seq	12Lean21Obesity	subcutaneous adipose tissue	Validation cohort

### Differentially Expressed Genes and Enrichment Investigation

A differential study was made on 34 lean and 38 obese GSE24883 and GSE25401 samples using the R software package limma. To filter DEGs, the limma software tool employs traditional Bayesian data analysis. For DEGs, the significant thresholds were established at an adjusted *P*-value of <0.05 and a log Fold Chang (logFC) larger than 1. The heatmap of DEGs was created using the heatmap software program. We used the R package cluster profile to undertake GO function enrichment analysis as well as KEGG enrichment analysis on associated genes, and we found three kinds of significantly enriched GO terms (*P* < 0.05) and considerably enriched pathways (*P* < 0.05) using metascope cluster analysis (http://metascape.org/gp/index,html).

### Construction of Protein-Protein Interaction (PPI)-Network

In the sting database (https://www.string-db.org/), we utilized the screened differential genes to create a PPI network. The interaction score for the PPI network's minimum requirement is set at 0.4. Simultaneously, while constructing a PPI network, we conceal solitary points that are not connected.

### Random Forest Screening for DEGs

For the DEGs, the Random Forest software tool was utilized to create a random forest model. Firstly, the average model inaccuracy rate for all genes was estimated using out-of-band data. The optimal variable value for the binary tree in the node has been set to 6, and the best number of trees in the random forest was decided to be 500. The dimensional effect size from the random forest model then was determined using the diminishing accuracy approach (Gini coefficient method). For the ensuing model development, illness genetic factors with an essential point larger than 1.2 were picked. The unstructured hierarchical groups of the 12 significant genes in the merging dataset were reclassified and a heatmap was produced using the freeware tool pheatmap.

### Modeling of an Artificial Neural Network

For neural network-based training, the GSE24883 and GSE25401 merging datasets were used. The R software package neural net has been used to develop a deep learning model of the main variables after the data was standardized to the maximum and lowest values. The model parameters for constructing an obese classification model using the collected gene weight information were set at four hidden layers. The illness classification score was calculated using the sum of the weight scores scaled by the differential expression of the key genes in this model. The validation outcomes of AUC classification results were then calculated using the pROC software tool.

### Evaluation of AUC

The validity of the categorization score model of slim and obese samples is evaluated using the following data sets (the merging dataset of GSE156906 and GSE159924). To check the classification efficiency, use the proc software tool to build the ROC curve for each and compute the area under the Curve. Simultaneously, the appropriate ROC curve threshold was determined, as well as the specificity and sensitivity of categorizing obese and healthy samples under this threshold.

### Estimation of the Immune Landscape and Correlation Test

Using the R package “complot” with 1000 permutations, CIBERSORT (https://cibersortx.stanford.edu/) has been used to infer the 22 immune-cell values in the obese cohort by analyzing the proportion of patients with the transcription of Leukocyte signature matrix (LM22) core genes. Cases with a CIBERSORT result of *P* < 0.05 were selected for the following analysis. Violin plots were constructed in R using the “vioplot” package to show the differences in immune-cell infiltration between the two groups. The association between the found gene indication and the quantity of invading immune cells was investigated using Spearman's correlation research in R. The charm method of the “ggplot2” package was used to depict the resulting correlations.

## Result

### Identification of DEGs

**Figure 2:**
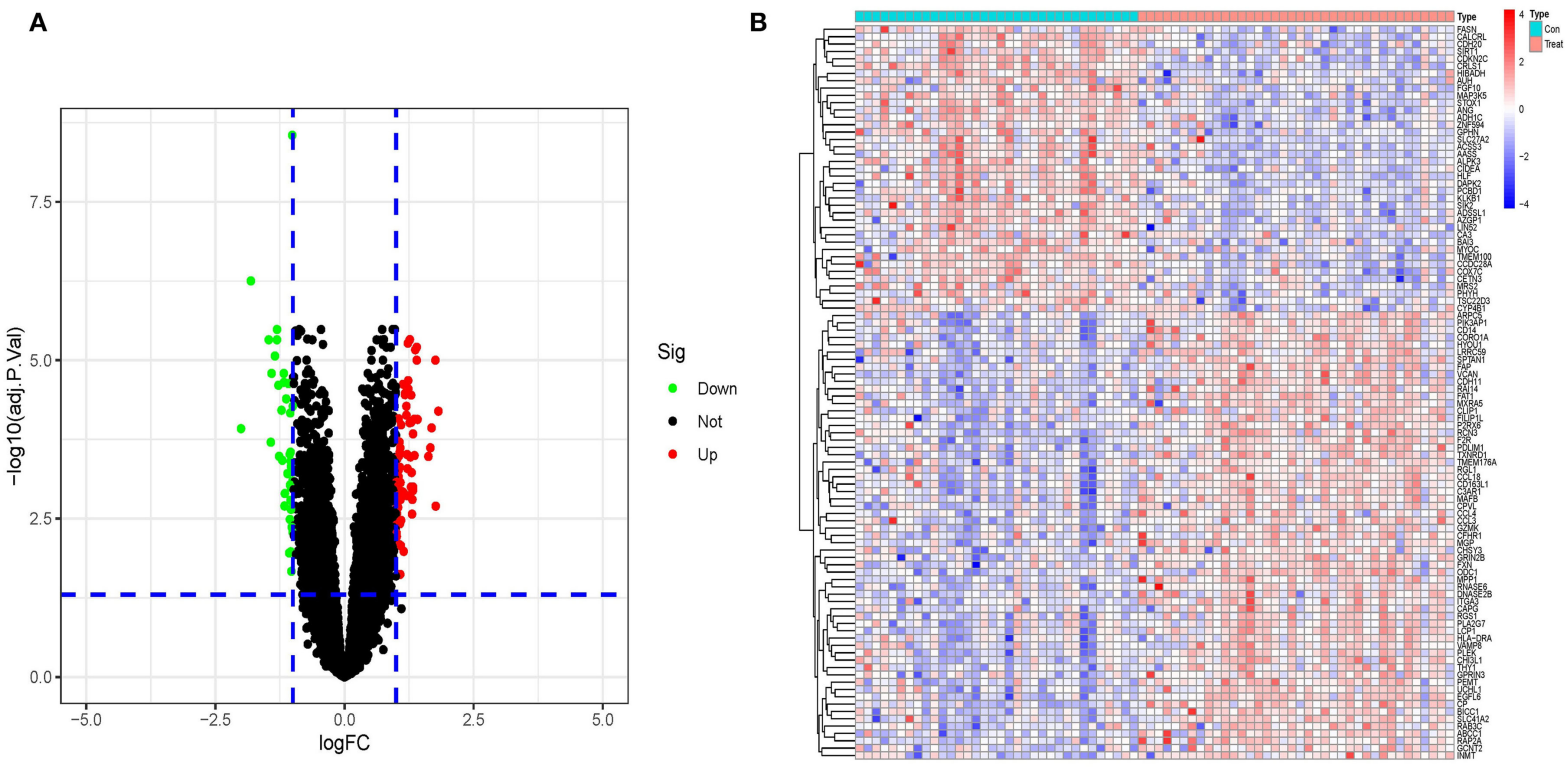
**(A)** A volcano plot representing the findings of differential expression investigation. The remaining functional genes are shown by the black dots. **(B)** A DEG heatmap. The graph's hues, which range from red to green, represent high to low expressiveness. The red band in the top half of the heatmap represents illness samples, whereas the blue band represents normal samples.

The Bayesian test was utilized to discover DEGs between obese chip dataset samples and lean control samples using the limma program. The DEGs' findings are depicted in the volcano diagram (Figure 2A) as well as the heatmap (Figure 2B). The search found 113 significant DEGs associated with obesity depending on fold change values of >1 as well as a significance threshold of *P* < 0.05 (Supplementary File 1).

### Metascape Analysis of DEGs

**Figure 3:**
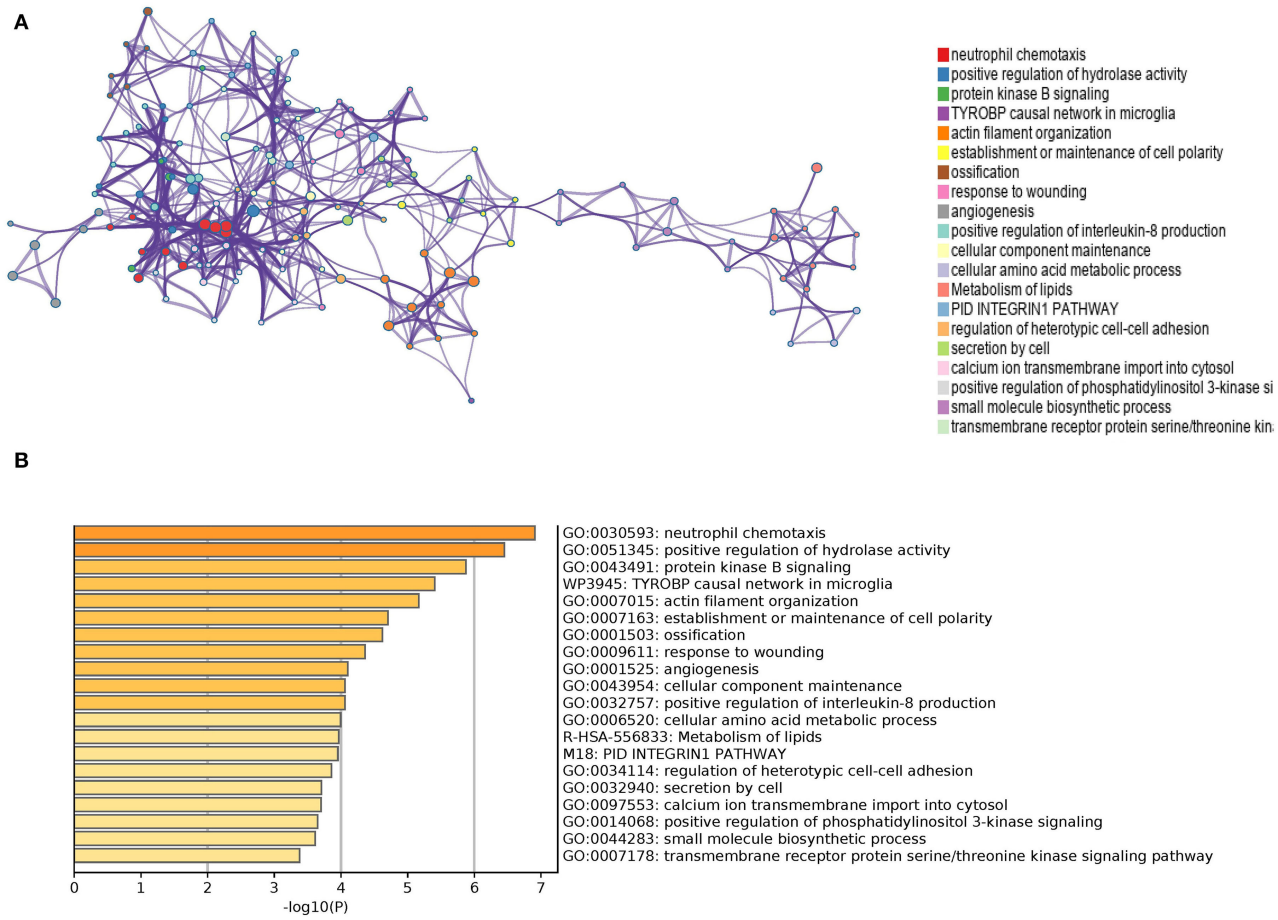
**(A)** An enhanced terms network. Cluster-ID is used to color the notes, and notes with the same cluster-ID are often closer to one other. **(B)** *P*-Value-colored bar graph of enhanced phrases across DEGs lists.

The matescape database was used to enrich and evaluate differential genes. GO Biological Processes, KEGG Pathway, Canonical Pathways, Cell Type Signatures, Reactome Gene Sets, CORUM, TRUST, DisGeNET, PaGenBase, Transcription Factor Targets, WikiPathways, PANTHER Pathway, and COVID were used to enrich the DEGs list using pathway and process enrichment investigation. The enrichment background was made up of every gene in the genome. Terms having a *p*-value < 0.01, a baseline count of 3, and contributing factors more than 1.5 (the maximum enhancement is the proportion between the known numbers and the counts anticipated by chance) are gathered and classified depending on membership commonalities. The top 20 words from the matescape enrichment analysis are shown in Figures 3A,B. Supplement File 2 contains the findings of the route and process enrichment study.

### Enrichment Analysis in Samples From Obese Patients and Lean People

**Figure 4:**
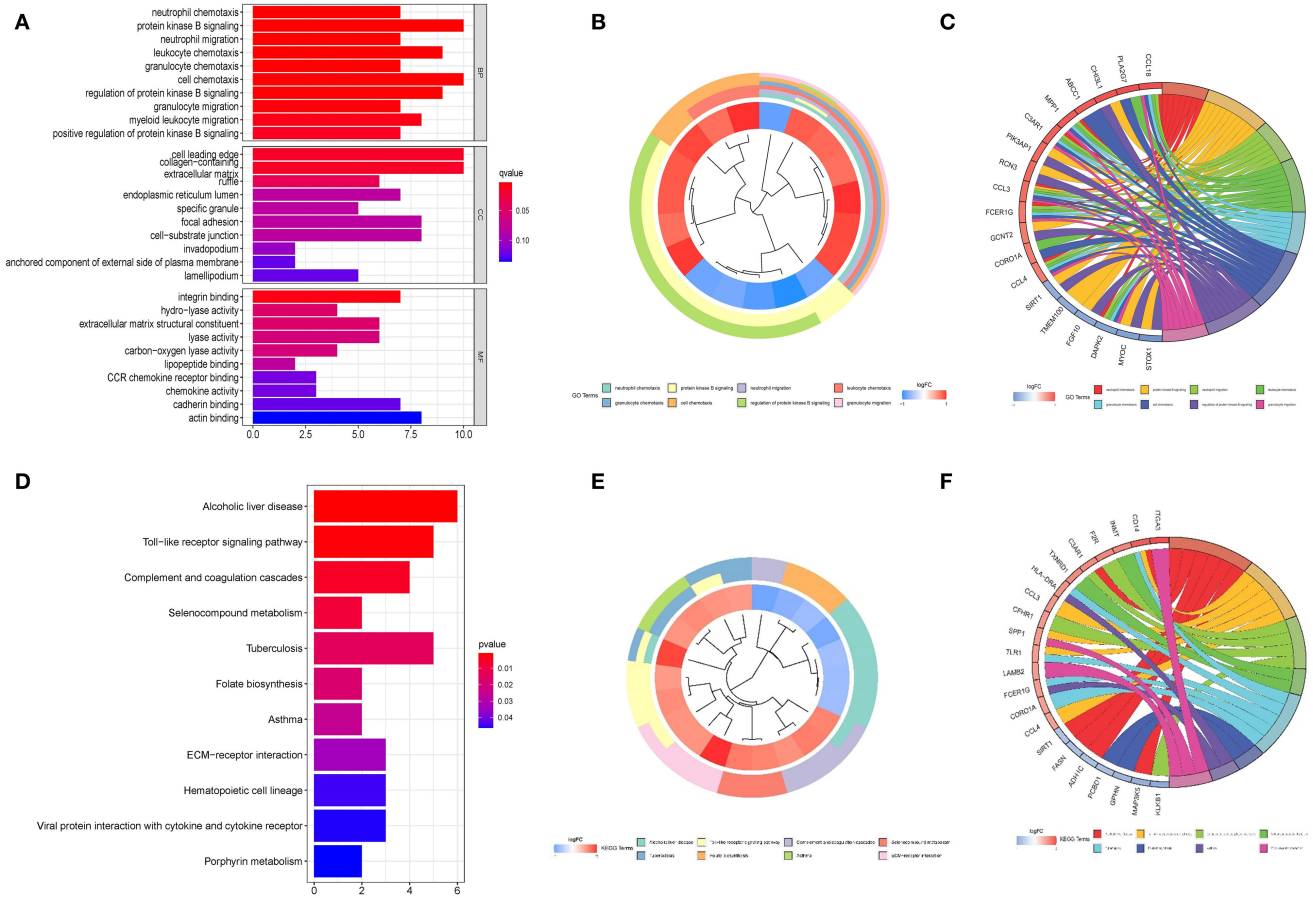
Graph depicting the findings of the enrichment analysis. **(A)** GO enrichment findings in a bar graph. The *z*-score is shown on the *x*-axis, while the log 10 (adj *P*) values are represented on the *y*-axis. **(B)** Gene clustering circle: the inner circle indicates DEGs, the red circle represents up-regulated genes, the blue circle indicates down-regulated genes, and the outside circle represents GO keywords. **(C)** GO enrichment ring plot. The DEGs are shown on the left, with the red gene band indicating upregulation and the blue gene band indicating downregulation. The right-hand band, which is colored differently, indicates several GO concepts. The gene's inclusion in the GO word is shown by the connecting line. **(D)** KEGG pathway enrichment findings in a bubble chart. The z-score is shown on the x-axis, while the log 10 (adj *P*) value is represented on the y-axis. A KEGG pathway is represented by a bubble, the size of which indicates the number of genes in the route. The route enrichment findings in the figure with a log 10 (adj *P*) > 1.3 (*P* < 0.05) are highlighted and listed in the table. **(E)** Gene clustering circle: the inner circle indicates DEGs, the red circle represents up-regulated genes, the blue circle indicates down-regulated genes, and the outside circle represents KEGG terms. **(F)** KEGG pathway enrichment ring plot. The DEGs are shown on the left side, with red gene bands indicating upregulation and blue gene bands indicating downregulation. Distinct colored bands on the right-hand side symbolize different paths. The gene's involvement in the route is shown by the connecting line.

The cluster profile software was used to conduct GO enrichment analysis on the 113 noteworthy DEGs. The Benjamini–Hochberg correction technique was applied, with the *P* and *Q* levels set at 0.05 and 0.05, respectively. We conducted compression on the GO enrichment words and excluded phrases with a gene overlap of >0.75 to prevent repetition in the GO enrichment findings. The findings of 3 areas of GO enrichment are shown in Figure 4. Figure 4A displays the GO enrichment findings for all three categories (only the –log10 (adj *P*) >5 GO terms are presented). Protein kinase B signaling, leukocyte chemotaxis, cell chemotaxis, modulation of protein kinase B signaling, and myeloid leukocyte migration are among the associated biological processes implicated in obesity, according to the findings. Cell leading edge and collagen-containing cellular components are involved. Integral interaction and other critical activities were among the molecular functionalities. Parts of the GO enriched words and the key DEGs implicated are shown in Figures 4B,C. On the DEGs, we also ran a KEGG pathway enrichment analysis. Figures 4D–F demonstrate the findings of substantially enriched biological KEGG pathways implicated, as well as the accompanying DEGs.

### Random Forest Tree Screening

**Figure 5:**
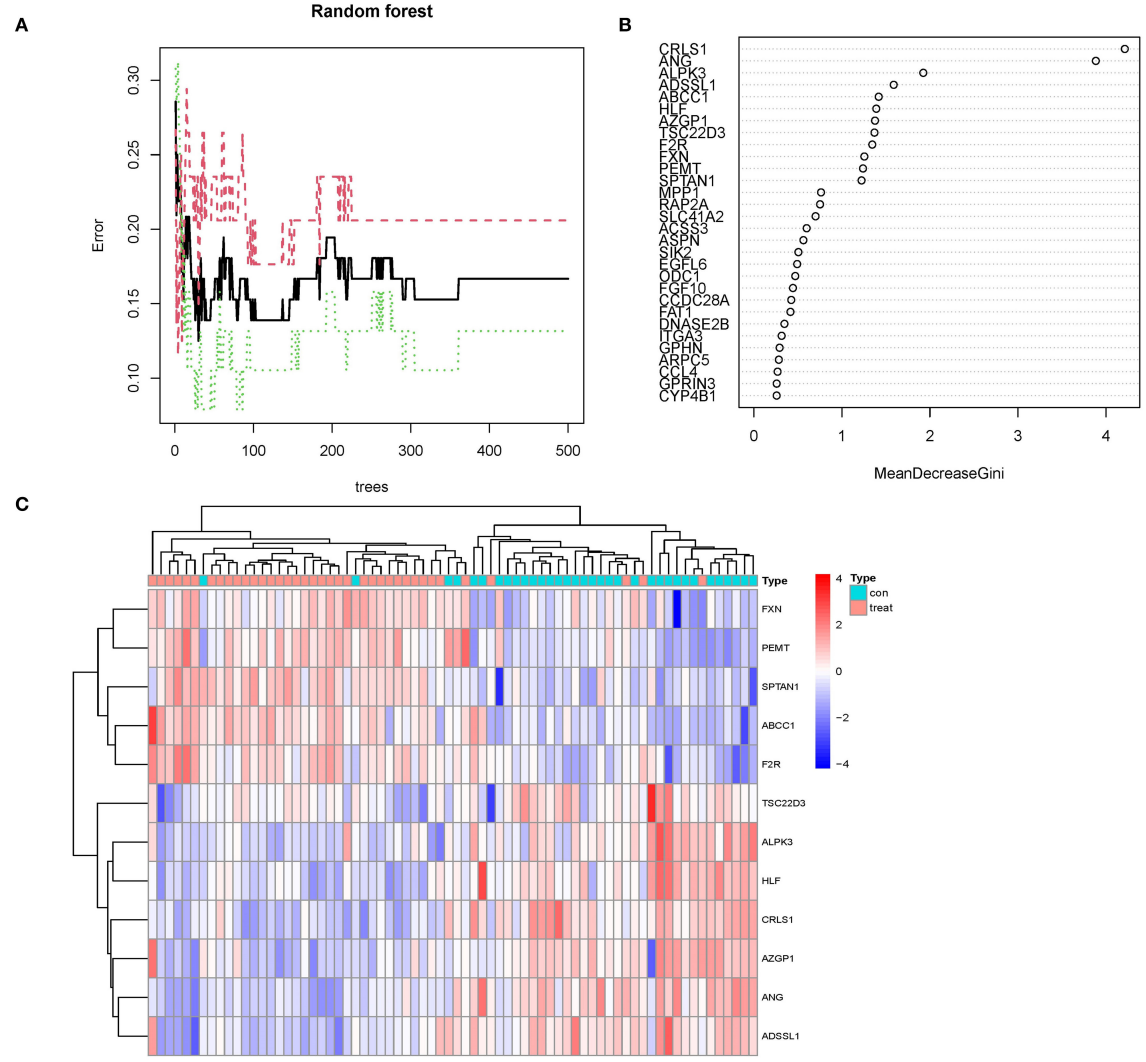
**(A)** The mistake rate is influenced by the number of selection trees. The amount of decision trees is shown on the *x-*axis, while the mistake rate is represented on the *y*-axis. **(B)** Random forest classifier results using the Gini coefficient approach. **(C)** Unsupervised clustering heatmap demonstrating the hierarchical clustering formed by the twelve significant genes created by the random forest in the GSE24881 and GSE25403 merging dataset. The red band on the upper portion of the heatmap suggests normal samples, while the blue band indicates obesity disorder samples. Red color demonstrates genes with elevated expression in the samples, the blue color implies genes with low or undetectable in the samples.

The random forest algorithm received the 113 DEGs. We did a recurrent random forest categorization for all possible values among the 1–113 factors and estimated the mean error rate of the model to determine the ideal parameter mtry (that is, to describe the best number of factors for the binary trees inside the nodes). As the variable number's argument, we picked 12. The set of variables was kept to a minimum, and out-of-band error was kept to an absolute minimum. We chose 500 trees as the variable of the final model based on the association plot between both the model uncertainties and the number of selection trees (Figure 5A), which demonstrated a steady error. The variable relevance of the output findings (Gini coefficient method) was assessed in the context of decreasing accuracy and decreasing mean square error throughout the construction of the random forest model (see Supplementary File 3 for the important output results). The potential genes for further investigation were then identified as twelve DEGs with a significance larger than 1.2. Figure 5B demonstrates that ALPK3, ADSSL1, ABCC1, ANG, CRLS1, HLF, AZGP1, TSC22D3, F2R, FXN, PEMT, and SPTAN1 were the most significant of the twelve variables. We used k-means unsupervised clustering to cluster the merging dataset using these twelve critical factors. The twelve genes might be utilized to differentiate between illness and normal samples, as shown in Figure 5C. FXN, SPTAN1, ABCC1, F2R, and PEMT are a group of genes with low or undetectable positive control and reach this point in treated samples. CRLS1, ANG, ALPK3, ADSSL1, HLF, AZGP1, and TSC22D3, on the other hand, belong to a different cluster, having a high level of expression in healthy samples but a low level of expression in ill samples.

### Constructing an Artificial Neural Network Model

**Figure 6:**
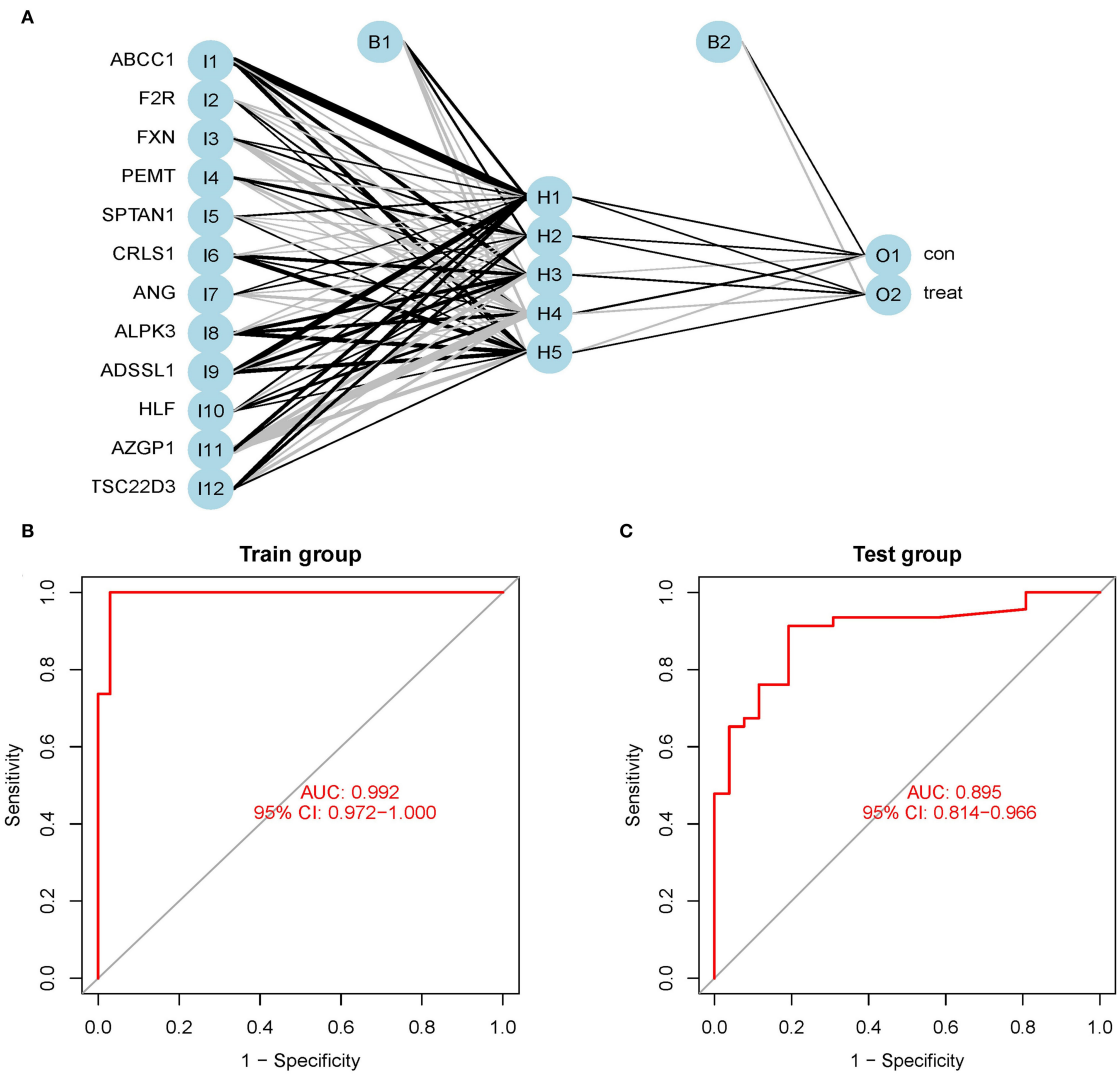
**(A)** Neural network visualization results. **(B)** The training group verifies the ROC curve findings (merge dataset of GSE24881 and GSE25403). **(C)** The testing group verifies the ROC curve findings (merge dataset of GSE156906 and GSE159924).

We utilized the GSE24881 and GSE25403 merging datasets to build an artificial neural network model using the neural net package. Data preparation was the initial phase, which was used to standardize the data. To segregate the magnification information before training the network, the min-max technique [0,1] was chosen and pushed. The maximum and lowest data values were normalized before the computation began, and the number of hidden layers was set to 5. There was no set guideline for how many layers and neurons to employ when choosing parameters. The number of neurons should be around two-thirds of the input layer size and one-third of the output layer size. As a result, the number of neurons parameter was adjusted to 12. A training data set and a validation set was created at random from the dataset. The objective of the training group was to figure out how much each candidate's DEG was worth. The validation set was utilized to test the model score's classification performance using the expression of genes and gene weight. The following is the formula for calculating the categorization score of the produced illness neural network model: neuraObesity = ∑(Gene Expression **×** Neural Network Weight) (Figure 6A). To create the neural network model, we utilize all of the data. The experimental group demonstrated that the model's area under the ROC curve (AUC) was near 1 (average AUC > 0.99), indicating that it was robust. To check that the area under the ROC curve (AUC) remains near 0.9, we examined the merged data sets of two more data sets, GSE156906 and GSE159924 (Figures 6B,C).

### Immune Landscape Associated With the Characteristics of Obesity Patients

**Figure 7:**
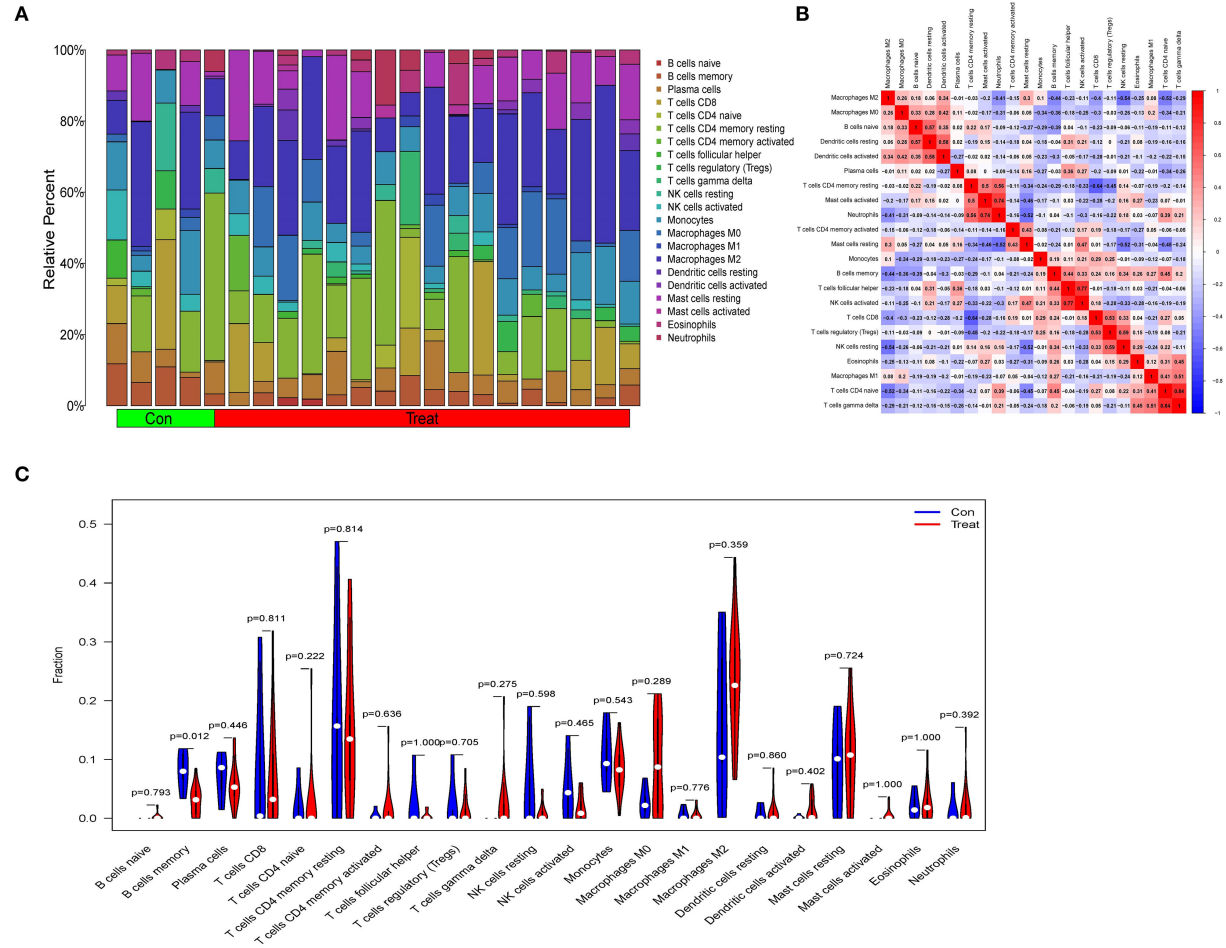
An examination of the immunological landscape of obesity. **(A)** Overview of predicted proportions of 22 immune-cell categories in con and treat groups using the CIBERSORT algorithm. **(B)** Correlation analysis of infiltrating immune cells. **(C)** Con and treat groups were compared on 22 immune-cell subtypes.

Immune-related networks were enhanced in the obese sample vs. in the lean category, according to functional enrichment analysis. Adipose tissue genomic information from the fusion dataset of GSE24881 and GSE25403 has been processed to investigate the immune landscape differences between obese patients and lean persons. The proportion of 22 distinct types of immune cells in the data was also calculated using the program CIBERSORTx. CIBERSORTx is an online tool that determines the relative quantity of immune adult tissues using a background subtraction algorithm. The location of 22 distinct immune cell types in obese and thin subjects is shown in Figure 7A. We compared the relationship between immune cells with Spearman's correlation analysis. The largest positive connection, *R* = 0.84, was found between T cells CD4 naïve and T cells gamma delta, whereas the strongest negative correlation, *R* = −0.64, was found between T cells CD4 memory resting and T cells CD8 (Figure 7B). In addition, the proportion of B cells with memory was significantly lower (P = 0.012) in the obese group than in the no-obese group (Figure 7C).

## Discussion

For the first time, we computed DEGs associated with obesity and discovered twelve key candidate DEGs using the classifier model in this work. We employed a neural network model to compute the anticipated weights of linked genes, create the neuraObesity classification model score, and test the model score's classification performance in 2 autonomous sample datasets. The AUC efficiency was outstanding, and it was discovered that neuraObesity had a high classification efficiency.

CRLS1 is a variation linked with insulin resistance, and adipose CRLS1 expression positively connects with insulin sensitivity among these twelve genes. By reducing the expression and activity of ATF3, CRLS1 reduces insulin resistance, hepatic steatosis, inflammation, and fibrosis during the pathological phase of non-alcoholic steatohepatitis (NASH) (10, 11). The angiotensin-angiotensin system is a critical regulator of metabolism, with the angiotensin 1-7 (ANG 1-7) peptide having positive effects. Treatment with ANG 1-7 lowered body weight, increased thermogenesis, and improved glucose homeostasis without changing food intake. Paternal inflammation-induced metabolic abnormalities in children are linked to ANG-mediated synthesis of 5'-tsRNAs in sperm, and offspring of inflamed fathers have metabolic diseases such as glucose intolerance and obesity (12, 13).

ABCC1 is a protein found in human adipocytes. ABCC1 mRNA is increased in adult adipose tissue, while tissue plasma cortisol concentrations are continuously low (14, 15). In the epidemic of obesity, AZGP1 is implicated in polygenic traits and age-dependent alterations in the genetic regulation of obesity. Reduced AZGP1 expression resulted in a considerable increase in lipogenic gene expression, resulting in increased serum lipid in KD cells. By negatively regulating TNF-α, AZGP1 reduces the severity of Nonalcoholic fatty liver disease (NAFLD) by lowering inflammation, speeding lipolysis, boosting proliferation, and minimizing apoptosis. AZGP1 has been proposed as a potential new treatment target for NAFLD. Circulating AZGP1 has been linked to polycystic ovary syndrome (PCOS) and might be a significant adipokine in the onset and progression of PCOS. A large number of literatures have confirmed that PCOS is closely related to obesity and insulin resistance (16). AZGP1 might be used as a novel observational biomarker in the management of PCOS patients. AZGP1 levels in the blood are lower in women with PCOS, and AZGP1 could be a cytokine linked to insulin resistance in PCOS patients (17–21). Adipogenesis was aided by the coagulation factor II thrombin receptor (F2R), which encodes coagulation factor II. Obesity, T2D, steatosis, atherosclerosis, as well as osteoporosis are all metabolic disorders, and the gene F2R might be exploited as an adipogenic marker to give a possible target for understanding them. F2R was identified as a potentially relevant biomarker related to the polycystic ovarian syndrome as a result of the PCOS pathway network that was created (PCOS) (22, 23). PEMT is a tiny integral membrane protein that transforms phosphatidylethanolamine (PE) to phosphatidylcholine (PC). PEMT knockdown prevented lipid droplet formation, lowered triacylglycerol concentration, and decreased leptin release from adipocytes (24–26). Fat migration into the periphery of the vast lateral, gastrocnemius, as well as soleus muscles, was seen in all ADSSL1 myopathy patients, as were increased lipid droplets (27).

Interestingly, none of the following four genes (ALPK3, HLF, FXN, and SPTAN1) have been shown to be involved in obesity-related disorders. Familial cardiomyopathy may be caused by ALPK3 mutations. Cardiomyocytes missing ALPK3 may have abnormal calcium handling, offering useful insights into the molecular processes driving ALPK3-mediated cardiomyopathy (28). HIF-2 activates the production of hypoxia-inducible, lipid droplet-associated protein in renal CCCs, which preferentially enriches polyunsaturated lipids, the rate-limiting precursors for lipid peroxidation (HILPDA) (29). Friedreich's ataxia (FRDA) is a neurological illness with T2D as severe comorbidity caused by reduced expression of mitochondrial frataxin (FXN). Hyperlipidemia, impaired energy expenditure, insulin sensitivity, as well as higher plasma leptin are all shown in the FXN knock-in/knock-out (KIKO) mouse, which mimics T2D-like symptoms. In BAT, FXN deficiency causes mitochondrial ultrastructure disruption, oxygen consumption, and lipid buildup (30). SPTAN1 is a potential gene for ataxia and spastic paraplegia, and also the disruption of spectrin helices' interlinking might be a crucial aspect of the pathomechanism for the mutations (31).

The majority of research have shown that proinflammatory T lymphocytes and macrophages play a key role in insulin resistance (IR) induced by visceral adipose tissue inflammation (VAT) (32). The invasion and activation of immune cells define adipose tissue inflammation. Immune cells release cytokines and chemokines, which lead to chronic inflammation and exacerbate the metabolic pathway deterioration associated with obesity. In obese individuals, CD8 + and Th1 CD4 + T cells enter VAT and stimulate the release of proinflammatory cytokines by M1 macrophages, according to studies (33). B cells are capable of presenting antigens to T cells, secreting proinflammatory cytokines and pathogenic antibodies. Lipolysis products in VAT may activate B cells, causing them to produce proinflammatory mediators and causing systemic and local inflammation. Our findings also indicated that obese persons had more T cells and macrophages, although there was no substantial difference when compared to healthy people. This might be due to the research sample size being too small (34, 35).

The current research contained several flaws. First, we searched DEGs in the GEO database comparing fat tissues from obese patients and normal fat samples without subtyping obese individuals. Second, the clinical applicability of the random forest, as well as the artificial neural network joint diagnostic model for obesity, has to be further evaluated and externally verified. This information will be made available in future research.

Finally, our findings clearly showed that a combined random forest and artificial neural network obesity diagnostic model is acceptable for forecasting obesity occurrence in clinical practice.

## Data Availability Statement

The datasets used during the present study are available from GEO (https://www.ncbi.nlm.nih.gov/geo/) database.

## Author Contributions

CW is the corresponding author of the article who contributes the most. She designed the whole study. YZ and XX collaborated on data curation. Statistical analysis was carried out by FJ and CW. The original draft of the manuscript was completed by JY. CW revised the manuscript. All of the authors approved the final manuscript.

## Funding

This work was supported by the National Natural Science Foundation of China (Grant No. 82172539) and funded by the Nanjing Municipal Science and Technology Bureau (Grant number of 2019060002). The funding bodies had no role in the study design, data collection, analysis, and interpretation of data.

## Conflict of Interest

The authors declare that the research was conducted in the absence of any commercial or financial relationships that could be construed as a potential conflict of interest.

## Publisher's Note

All claims expressed in this article are solely those of the authors and do not necessarily represent those of their affiliated organizations, or those of the publisher, the editors and the reviewers. Any product that may be evaluated in this article, or claim that may be made by its manufacturer, is not guaranteed or endorsed by the publisher.

## Supplementary Material

Supplement File 1 113 significant DEGs related to obesity.

Supplement File 2 Results of path and process enrichment analysis.

Supplement File 3 Results of random forest classifier.
